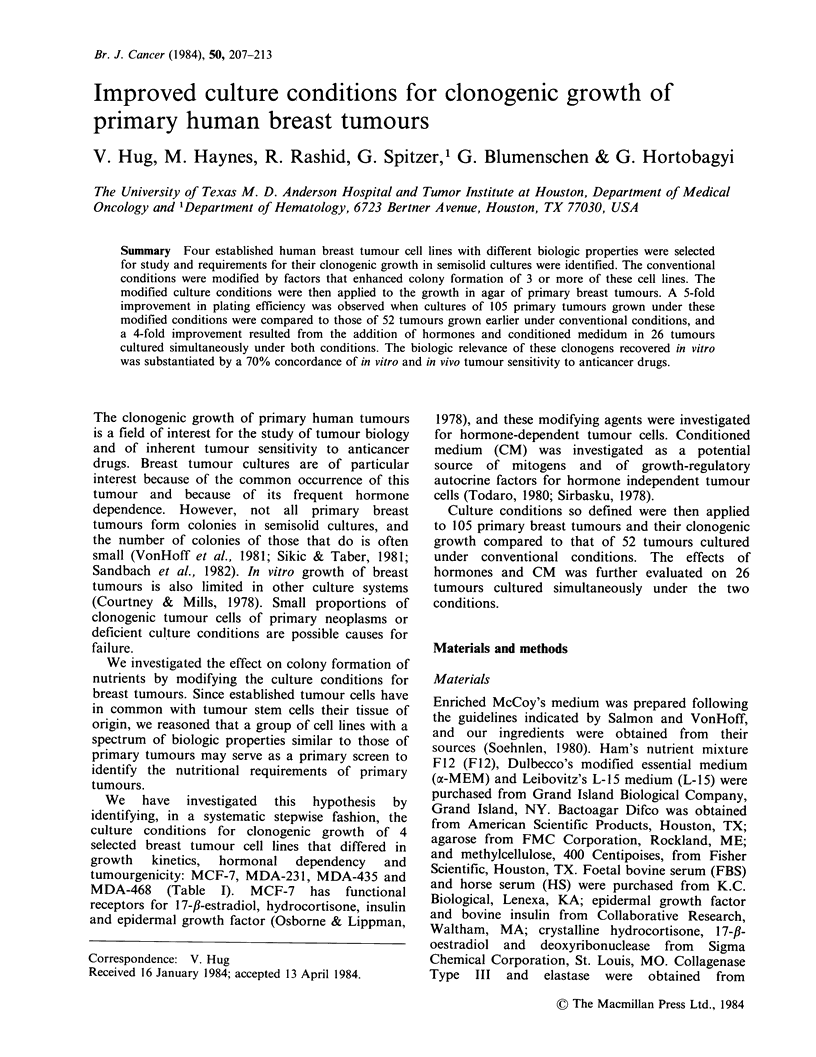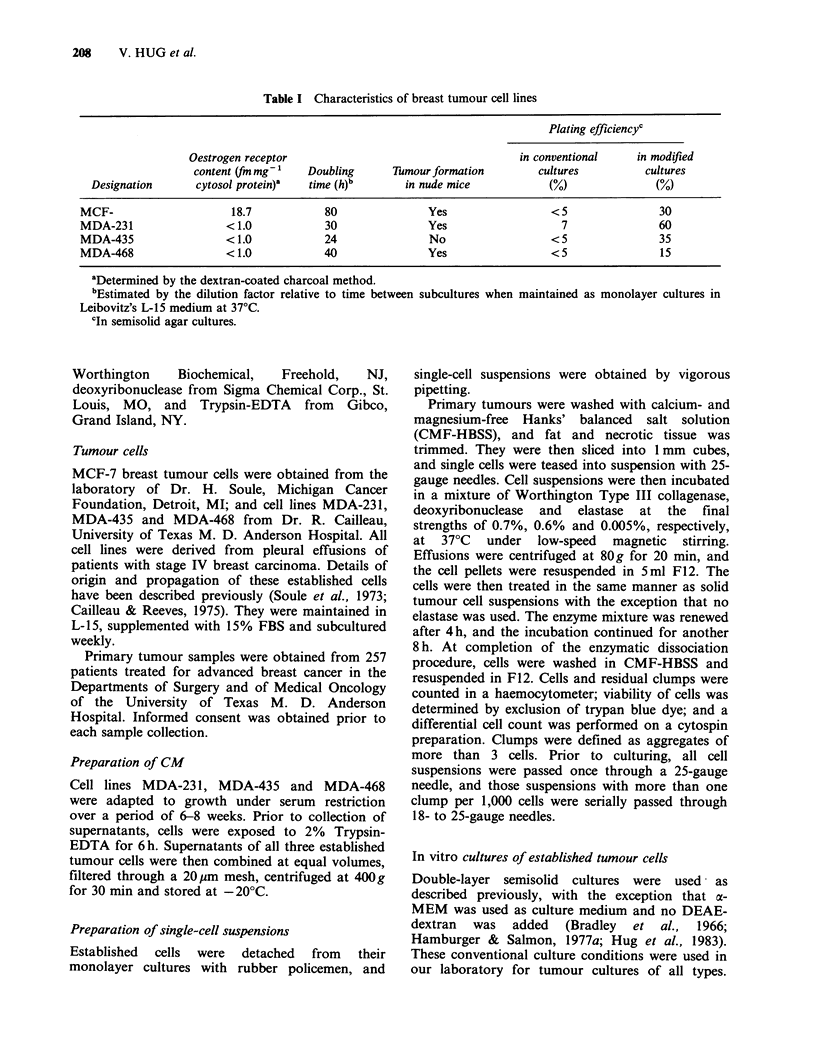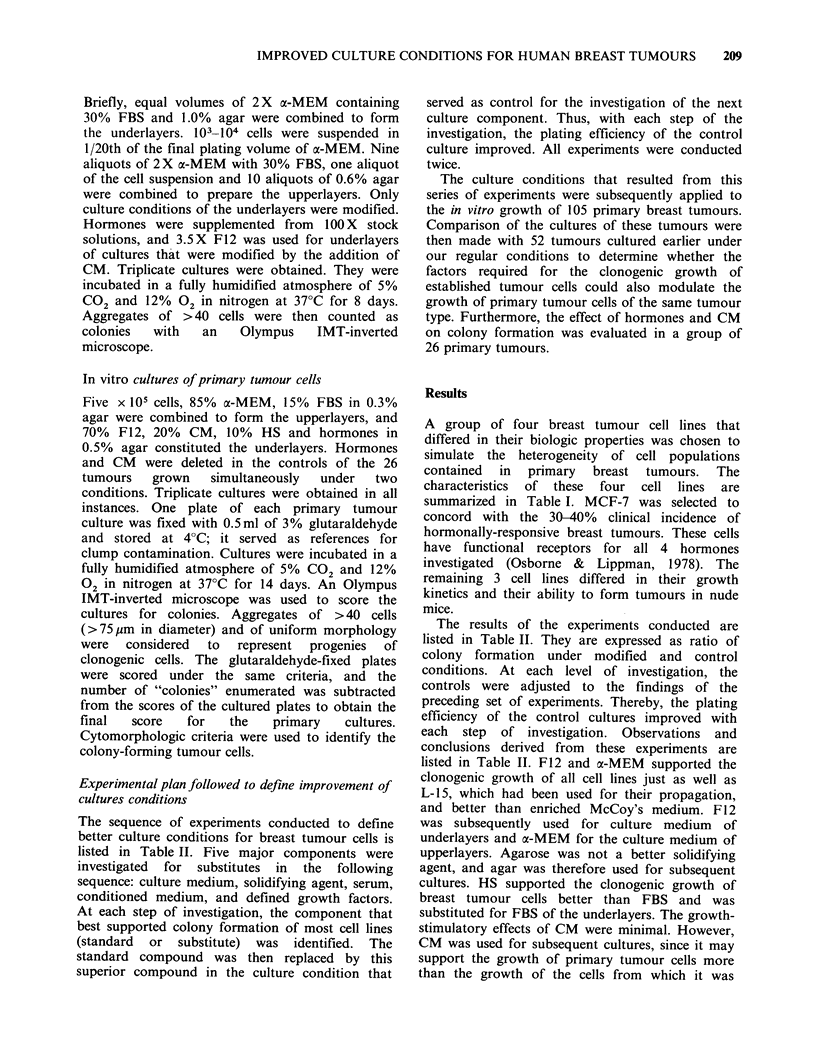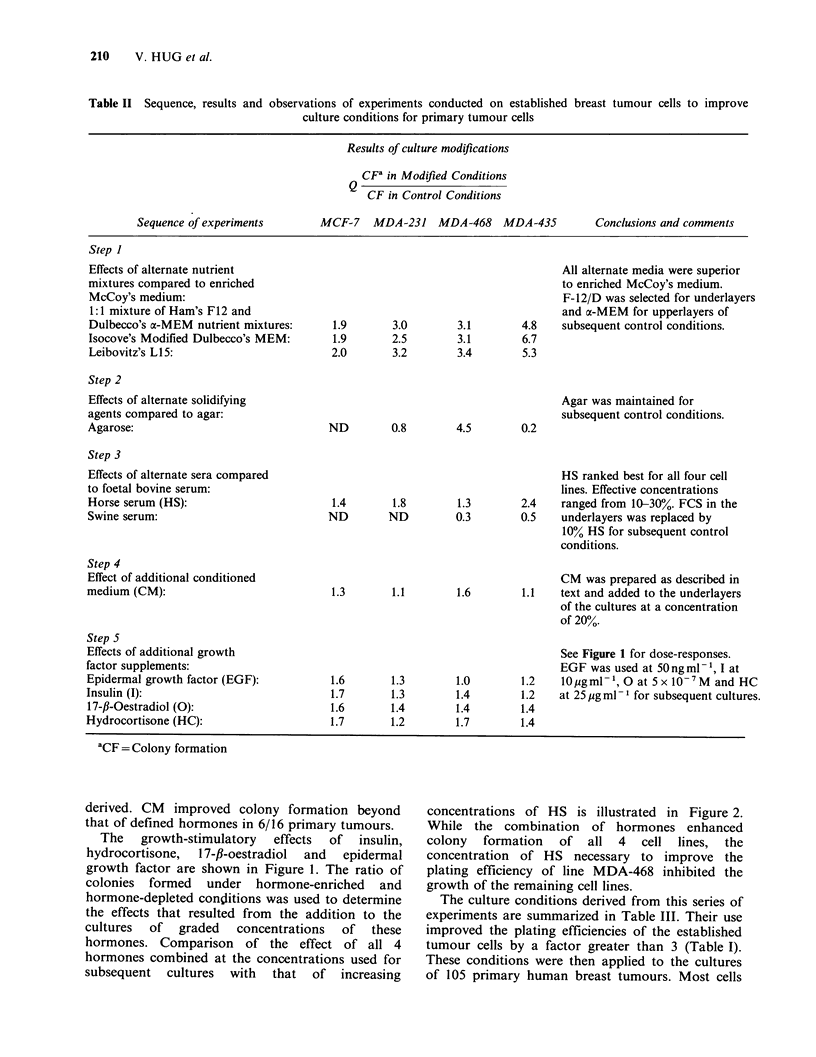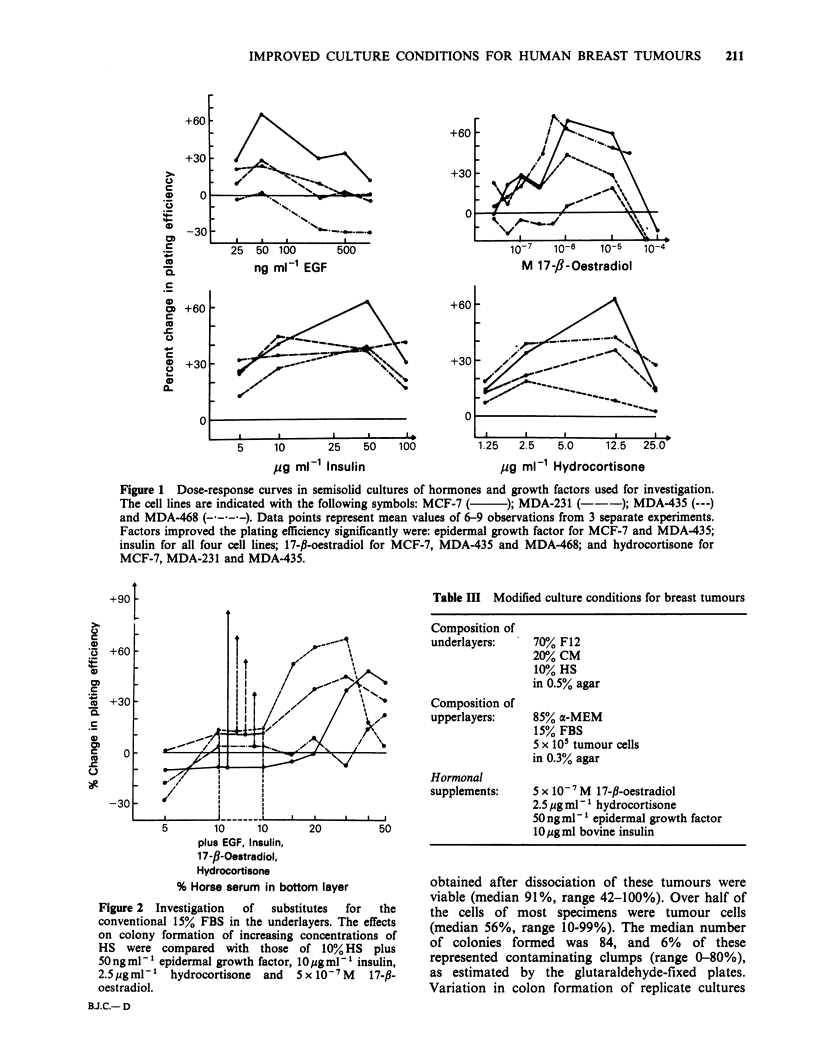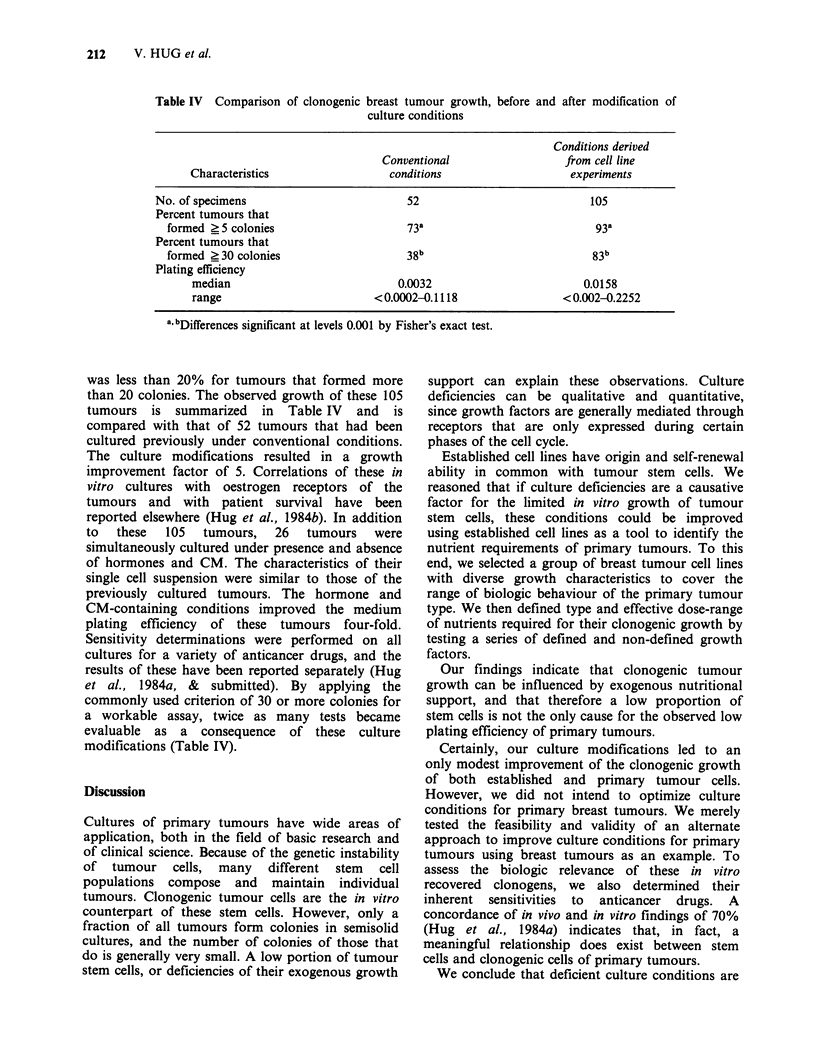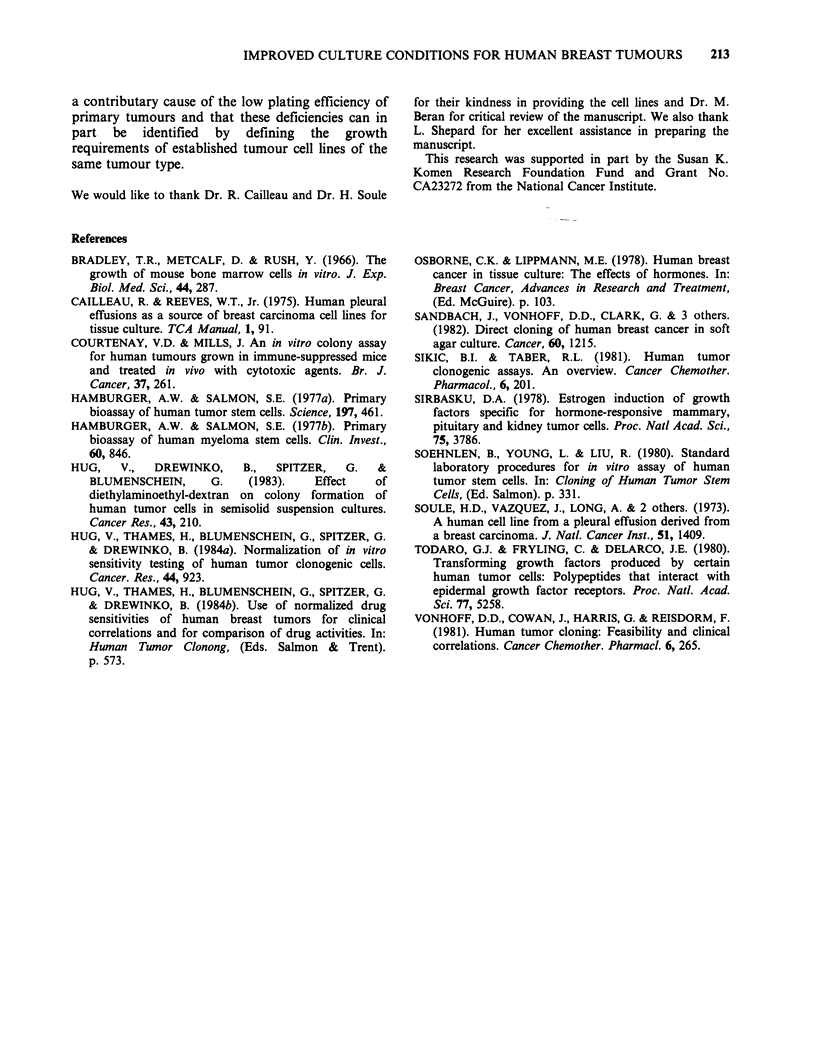# Improved culture conditions for clonogenic growth of primary human breast tumours.

**DOI:** 10.1038/bjc.1984.164

**Published:** 1984-08

**Authors:** V. Hug, M. Haynes, R. Rashid, G. Spitzer, G. Blumenschen, G. Hortobagyi

## Abstract

Four established human breast tumour cell lines with different biologic properties were selected for study and requirements for their clonogenic growth in semisolid cultures were identified. The conventional conditions were modified by factors that enhanced colony formation of 3 or more of these cell lines. The modified culture conditions were then applied to the growth in agar of primary breast tumours. A 5-fold improvement in plating efficiency was observed when cultures of 105 primary tumours grown under these modified conditions were compared to those of 52 tumours grown earlier under conventional conditions, and a 4-fold improvement resulted from the addition of hormones and conditioned medidum in 26 tumours cultured simultaneously under both conditions. The biologic relevance of these clonogens recovered in vitro was substantiated by a 70% concordance of in vitro and in vivo tumour sensitivity to anticancer drugs.


*Br. J. Cancer* (1984), **50**, 207–213

### Improved culture conditions for clonogenic growth of primary human breast tumours

V. Hug, M. Haynes, R. Rashid, G. Spitzer,[1] G. Blumenschen & G. Hortobagyi

*The University of Texas M. D. Anderson Hospital and Tumor Institute at Houston, Department of Medical Oncology and [1]Department of Hematology, 6723 Bertner Avenue, Houston, TX 77030, USA*

**Summary** Four established human breast tumour cell lines with different biologic properties were selected for study and requirements for their clonogenic growth in semisolid cultures were identified. The conventional conditions were modified by factors that enhanced colony formation of 3 or more of these cell lines. The modified culture conditions were then applied to the growth in agar of primary breast tumours. A 5-fold improvement in plating efficiency was observed when cultures of 105 primary tumours grown under these modified conditions were compared to those of 52 tumours grown earlier under conventional conditions, and a 4-fold improvement resulted from the addition of hormones and conditioned medidum in 26 tumours cultured simultaneously under both conditions. The biologic relevance of these clonogens recovered *in vitro* was substantiated by a 70% concordance of *in vitro* and *in vivo* tumour sensitivity to anticancer drugs.

The clonogenic growth of primary human tumours is a field of interest for the study of tumour biology and of inherent tumour sensitivity to anticancer drugs. Breast tumour cultures are of particular interest because of the common occurrence of this tumour and because of its frequent hormone dependence. However, not all primary breast tumours form colonies in semisolid cultures, and the number of colonies of those that do is often small (VonHoff *et al.,* 1981; Sikic & Taber, 1981; Sandbach *et al.,* 1982). *In vitro* growth of breast tumours is also limited in other culture systems (Courtney & Mills, 1978). Small proportions of clonogenic tumour cells of primary neoplasms or deficient culture conditions are possible causes for failure.

We investigated the effect on colony formation of nutrients by modifying the culture conditions for breast tumours. Since established tumour cells have in common with tumour stem cells their tissue of origin, we reasoned that a group of cell lines with a spectrum of biologic properties similar to those of primary tumours may serve as a primary screen to identify the nutritional requirements of primary tumours.

We have investigated this hypothesis by identifying, in a systematic stepwise fashion, the culture conditions for clonogenic growth of 4 selected breast tumour cell lines that differed in growth kinetics, hormonal dependency and tumourgenicity: MCF-7, MDA-231, MDA-435 and MDA-468 (Table I). MCF-7 has functional receptors for 17-$\beta$-estradiol, hydrocortisone, insulin and epidermal growth factor (Osborne & Lippman, 1978), and these modifying agents were investigated for hormone-dependent tumour cells. Conditioned medium (CM) was investigated as a potential source of mitogens and of growth-regulatory autocrine factors for hormone independent tumour cells (Todaro, 1980; Sirbasku, 1978).

Culture conditions so defined were then applied to 105 primary breast tumours and their clonogenic growth compared to that of 52 tumours cultured under conventional conditions. The effects of hormones and CM was further evaluated on 26 tumours cultured simultaneously under the two conditions.

Correspondence: V. Hug
Received 16 January 1984; accepted 13 April 1984.

### Materials and methods

### *Materials*

Enriched McCoy's medium was prepared following the guidelines indicated by Salmon and VonHoff, and our ingredients were obtained from their sources (Soehnlen, 1980). Ham's nutrient mixture F12 (F12), Dulbecco's modified essential medium ($\alpha$-MEM) and Leibovitz's L-15 medium (L-15) were purchased from Grand Island Biological Company, Grand Island, NY. Bactoagar Difco was obtained from American Scientific Products, Houston, TX; agarose from FMC Corporation, Rockland, ME; and methylcellulose, 400 Centipoises, from Fisher Scientific, Houston, TX. Foetal bovine serum (FBS) and horse serum (HS) were purchased from K.C. Biological, Lenexa, KA; epidermal growth factor and bovine insulin from Collaborative Research, Waltham, MA; crystalline hydrocortisone, 17-$\beta$-oestradiol and deoxyribonuclease from Sigma Chemical Corporation, St. Louis, MO. Collagenase Type III and elastase were obtained from

© The Macmillan Press Ltd., 1984

**Table I** Characteristics of breast tumour cell lines

| Designation | Oestrogen receptor content (fm mg$^{-1}$ cytosol protein)[a] | Doubling time (h)[b] | Tumour formation in nude mice | Plating efficiency[c]: in conventional cultures (%) | in modified cultures (%) |
|---|---|---|---|---|---|
| MCF- | 18.7 | 80 | Yes | <5 | 30 |
| MDA-231 | <1.0 | 30 | Yes | 7 | 60 |
| MDA-435 | <1.0 | 24 | No | <5 | 35 |
| MDA-468 | <1.0 | 40 | Yes | <5 | 15 |

[a]Determined by the dextran-coated charcoal method.
[b]Estimated by the dilution factor relative to time between subcultures when maintained as monolayer cultures in Leibovitz's L-15 medium at 37°C.
[c]In semisolid agar cultures.

Worthington Biochemical, Freehold, NJ, deoxyribonuclease from Sigma Chemical Corp., St. Louis, MO, and Trypsin-EDTA from Gibco, Grand Island, NY.

*Tumour cells*

MCF-7 breast tumour cells were obtained from the laboratory of Dr. H. Soule, Michigan Cancer Foundation, Detroit, MI; and cell lines MDA-231, MDA-435 and MDA-468 from Dr. R. Cailleau, University of Texas M. D. Anderson Hospital. All cell lines were derived from pleural effusions of patients with stage IV breast carcinoma. Details of origin and propagation of these established cells have been described previously (Soule *et al.*, 1973; Cailleau & Reeves, 1975). They were maintained in L-15, supplemented with 15% FBS and subcultured weekly.

Primary tumour samples were obtained from 257 patients treated for advanced breast cancer in the Departments of Surgery and of Medical Oncology of the University of Texas M. D. Anderson Hospital. Informed consent was obtained prior to each sample collection.

*Preparation of CM*

Cell lines MDA-231, MDA-435 and MDA-468 were adapted to growth under serum restriction over a period of 6–8 weeks. Prior to collection of supernatants, cells were exposed to 2% Trypsin-EDTA for 6 h. Supernatants of all three established tumour cells were then combined at equal volumes, filtered through a 20 $\mu$m mesh, centrifuged at 400 *g* for 30 min and stored at −20°C.

*Preparation of single-cell suspensions*

Established cells were detached from their monolayer cultures with rubber policemen, and single-cell suspensions were obtained by vigorous pipetting.

Primary tumours were washed with calcium- and magnesium-free Hanks' balanced salt solution (CMF-HBSS), and fat and necrotic tissue was trimmed. They were then sliced into 1 mm cubes, and single cells were teased into suspension with 25-gauge needles. Cell suspensions were then incubated in a mixture of Worthington Type III collagenase, deoxyribonuclease and elastase at the final strengths of 0.7%, 0.6% and 0.005%, respectively, at 37°C under low-speed magnetic stirring. Effusions were centrifuged at 80 *g* for 20 min, and the cell pellets were resuspended in 5 ml F12. The cells were then treated in the same manner as solid tumour cell suspensions with the exception that no elastase was used. The enzyme mixture was renewed after 4 h, and the incubation continued for another 8 h. At completion of the enzymatic dissociation procedure, cells were washed in CMF-HBSS and resuspended in F12. Cells and residual clumps were counted in a haemocytometer; viability of cells was determined by exclusion of trypan blue dye; and a differential cell count was performed on a cytospin preparation. Clumps were defined as aggregates of more than 3 cells. Prior to culturing, all cell suspensions were passed once through a 25-gauge needle, and those suspensions with more than one clump per 1,000 cells were serially passed through 18- to 25-gauge needles.

In vitro *cultures of established tumour cells*

Double-layer semisolid cultures were used as described previously, with the exception that α-MEM was used as culture medium and no DEAE-dextran was added (Bradley *et al.*, 1966; Hamburger & Salmon, 1977*a*; Hug *et al.*, 1983). These conventional culture conditions were used in our laboratory for tumour cultures of all types.

Briefly, equal volumes of 2X α-MEM containing 30% FBS and 1.0% agar were combined to form the underlayers. $10^3$–$10^4$ cells were suspended in 1/20th of the final plating volume of α-MEM. Nine aliquots of 2X α-MEM with 30% FBS, one aliquot of the cell suspension and 10 aliquots of 0.6% agar were combined to prepare the upperlayers. Only culture conditions of the underlayers were modified. Hormones were supplemented from 100X stock solutions, and 3.5X F12 was used for underlayers of cultures that were modified by the addition of CM. Triplicate cultures were obtained. They were incubated in a fully humidified atmosphere of 5% $CO_2$ and 12% $O_2$ in nitrogen at 37°C for 8 days. Aggregates of >40 cells were then counted as colonies with an Olympus IMT-inverted microscope.

*In* vitro *cultures of primary tumour cells*

Five $\times 10^5$ cells, 85% α-MEM, 15% FBS in 0.3% agar were combined to form the upperlayers, and 70% F12, 20% CM, 10% HS and hormones in 0.5% agar constituted the underlayers. Hormones and CM were deleted in the controls of the 26 tumours grown simultaneously under two conditions. Triplicate cultures were obtained in all instances. One plate of each primary tumour culture was fixed with 0.5 ml of 3% glutaraldehyde and stored at 4°C; it served as references for clump contamination. Cultures were incubated in a fully humidified atmosphere of 5% $CO_2$ and 12% $O_2$ in nitrogen at 37°C for 14 days. An Olympus IMT-inverted microscope was used to score the cultures for colonies. Aggregates of >40 cells (>75 μm in diameter) and of uniform morphology were considered to represent progenies of clonogenic cells. The glutaraldehyde-fixed plates were scored under the same criteria, and the number of "colonies" enumerated was subtracted from the scores of the cultured plates to obtain the final score for the primary cultures. Cytomorphologic criteria were used to identify the colony-forming tumour cells.

*Experimental plan followed to define improvement of cultures conditions*

The sequence of experiments conducted to define better culture conditions for breast tumour cells is listed in Table II. Five major components were investigated for substitutes in the following sequence: culture medium, solidifying agent, serum, conditioned medium, and defined growth factors. At each step of investigation, the component that best supported colony formation of most cell lines (standard or substitute) was identified. The standard compound was then replaced by this superior compound in the culture condition that served as control for the investigation of the next culture component. Thus, with each step of the investigation, the plating efficiency of the control culture improved. All experiments were conducted twice.

The culture conditions that resulted from this series of experiments were subsequently applied to the *in vitro* growth of 105 primary breast tumours. Comparison of the cultures of these tumours were then made with 52 tumours cultured earlier under our regular conditions to determine whether the factors required for the clonogenic growth of established tumour cells could also modulate the growth of primary tumour cells of the same tumour type. Furthermore, the effect of hormones and CM on colony formation was evaluated in a group of 26 primary tumours.

### Results

A group of four breast tumour cell lines that differed in their biologic properties was chosen to simulate the heterogeneity of cell populations contained in primary breast tumours. The characteristics of these four cell lines are summarized in Table I. MCF-7 was selected to concord with the 30–40% clinical incidence of hormonally-responsive breast tumours. These cells have functional receptors for all 4 hormones investigated (Osborne & Lippman, 1978). The remaining 3 cell lines differed in their growth kinetics and their ability to form tumours in nude mice.

The results of the experiments conducted are listed in Table II. They are expressed as ratio of colony formation under modified and control conditions. At each level of investigation, the controls were adjusted to the findings of the preceding set of experiments. Thereby, the plating efficiency of the control cultures improved with each step of investigation. Observations and conclusions derived from these experiments are listed in Table II. F12 and α-MEM supported the clonogenic growth of all cell lines just as well as L-15, which had been used for their propagation, and better than enriched McCoy's medium. F12 was subsequently used for culture medium of underlayers and α-MEM for the culture medium of upperlayers. Agarose was not a better solidifying agent, and agar was therefore used for subsequent cultures. HS supported the clonogenic growth of breast tumour cells better than FBS and was substituted for FBS of the underlayers. The growth-stimulatory effects of CM were minimal. However, CM was used for subsequent cultures, since it may support the growth of primary tumour cells more than the growth of the cells from which it was

**Table II** Sequence, results and observations of experiments conducted on established breast tumour cells to improve culture conditions for primary tumour cells

| Sequence of experiments | Results of culture modifications $Q\ \frac{CF^{a}\ \text{in Modified Conditions}}{CF\ \text{in Control Conditions}}$ MCF-7 | MDA-231 | MDA-468 | MDA-435 | Conclusions and comments |
|---|---|---|---|---|---|
| *Step 1* | | | | | |
| Effects of alternate nutrient mixtures compared to enriched McCoy's medium: | | | | | All alternate media were superior to enriched McCoy's medium. F-12/D was selected for underlayers and α-MEM for upperlayers of subsequent control conditions. |
| 1:1 mixture of Ham's F12 and Dulbecco's α-MEM nutrient mixtures: | 1.9 | 3.0 | 3.1 | 4.8 | |
| Isocove's Modified Dulbecco's MEM: | 1.9 | 2.5 | 3.1 | 6.7 | |
| Leibovitz's L15: | 2.0 | 3.2 | 3.4 | 5.3 | |
| *Step 2* | | | | | |
| Effects of alternate solidifying agents compared to agar: | | | | | Agar was maintained for subsequent control conditions. |
| Agarose: | ND | 0.8 | 4.5 | 0.2 | |
| *Step 3* | | | | | |
| Effects of alternate sera compared to foetal bovine serum: | | | | | HS ranked best for all four cell lines. Effective concentrations ranged from 10–30%. FCS in the underlayers was replaced by 10% HS for subsequent control conditions. |
| Horse serum (HS): | 1.4 | 1.8 | 1.3 | 2.4 | |
| Swine serum: | ND | ND | 0.3 | 0.5 | |
| *Step 4* | | | | | |
| Effect of additional conditioned medium (CM): | 1.3 | 1.1 | 1.6 | 1.1 | CM was prepared as described in text and added to the underlayers of the cultures at a concentration of 20%. |
| *Step 5* | | | | | |
| Effects of additional growth factor supplements: | | | | | See **Figure 1** for dose-responses. EGF was used at $50\,ng\,ml^{-1}$, I at $10\,\mu g\,ml^{-1}$, O at $5\times10^{-7}$ M and HC at $25\,\mu g\,ml^{-1}$ for subsequent cultures. |
| Epidermal growth factor (EGF): | 1.6 | 1.3 | 1.0 | 1.2 | |
| Insulin (I): | 1.7 | 1.3 | 1.4 | 1.2 | |
| 17-β-Oestradiol (O): | 1.6 | 1.4 | 1.4 | 1.4 | |
| Hydrocortisone (HC): | 1.7 | 1.2 | 1.7 | 1.4 | |

[a]CF = Colony formation derived. CM improved colony formation beyond that of defined hormones in 6/16 primary tumours.

The growth-stimulatory effects of insulin, hydrocortisone, 17-β-oestradiol and epidermal growth factor are shown in Figure 1. The ratio of colonies formed under hormone-enriched and hormone-depleted conditions was used to determine the effects that resulted from the addition to the cultures of graded concentrations of these hormones. Comparison of the effect of all 4 hormones combined at the concentrations used for subsequent cultures with that of increasing concentrations of HS is illustrated in Figure 2. While the combination of hormones enhanced colony formation of all 4 cell lines, the concentration of HS necessary to improve the plating efficiency of line MDA-468 inhibited the growth of the remaining cell lines.

The culture conditions derived from this series of experiments are summarized in Table III. Their use improved the plating efficiencies of the established tumour cells by a factor greater than 3 (Table I). These conditions were then applied to the cultures of 105 primary human breast tumours. Most cells

**Figure 1** Dose-response curves in semisolid cultures of hormones and growth factors used for investigation. The cell lines are indicated with the following symbols: MCF-7 (————); MDA-231 (— — —); MDA-435 (---) and MDA-468 (-·-·-·-). Data points represent mean values of 6–9 observations from 3 separate experiments. Factors improved the plating efficiency significantly were: epidermal growth factor for MCF-7 and MDA-435; insulin for all four cell lines; 17-β-oestradiol for MCF-7, MDA-435 and MDA-468; and hydrocortisone for MCF-7, MDA-231 and MDA-435.

**Figure 2** Investigation of substitutes for the conventional 15% FBS in the underlayers. The effects on colony formation of increasing concentrations of HS were compared with those of 10%HS plus $50\,ng\,ml^{-1}$ epidermal growth factor, $10\,\mu g\,ml^{-1}$ insulin, $2.5\,\mu g\,ml^{-1}$ hydrocortisone and $5\times10^{-7}$M 17-β-oestradiol.

**Table III** Modified culture conditions for breast tumours

| | |
|---|---|
| Composition of underlayers: | 70% F12<br>20% CM<br>10% HS<br>in 0.5% agar |
| Composition of upperlayers: | 85% α-MEM<br>15% FBS<br>$5\times10^5$ tumour cells<br>in 0.3% agar |
| *Hormonal* supplements: | $5\times10^{-7}$M 17-β-oestradiol<br>$2.5\,\mu g\,ml^{-1}$ hydrocortisone<br>$50\,ng\,ml^{-1}$ epidermal growth factor<br>$10\,\mu g$ ml bovine insulin |

obtained after dissociation of these tumours were viable (median 91%, range 42–100%). Over half of the cells of most specimens were tumour cells (median 56%, range 10-99%). The median number of colonies formed was 84, and 6% of these represented contaminating clumps (range 0–80%), as estimated by the glutaraldehyde-fixed plates. Variation in colon formation of replicate cultures

**Table IV** Comparison of clonogenic breast tumour growth, before and after modification of culture conditions

| Characteristics | *Conventional conditions* | *Conditions derived from cell line experiments* |
|---|---|---|
| No. of specimens | 52 | 105 |
| Percent tumours that formed ≧5 colonies | 73[a] | 93[a] |
| Percent tumours that formed ≧30 colonies | 38[b] | 83[b] |
| Plating efficiency | | |
| median | 0.0032 | 0.0158 |
| range | <0.0002–0.1118 | <0.002–0.2252 |

[a, b]Differences significant at levels 0.001 by Fisher's exact test.

was less than 20% for tumours that formed more than 20 colonies. The observed growth of these 105 tumours is summarized in Table IV and is compared with that of 52 tumours that had been cultured previously under conventional conditions. The culture modifications resulted in a growth improvement factor of 5. Correlations of these *in vitro* cultures with oestrogen receptors of the tumours and with patient survival have been reported elsewhere (Hug *et al.*, 1984*b*). In addition to these 105 tumours, 26 tumours were simultaneously cultured under presence and absence of hormones and CM. The characteristics of their single cell suspension were similar to those of the previously cultured tumours. The hormone and CM-containing conditions improved the medium plating efficiency of these tumours four-fold. Sensitivity determinations were performed on all cultures for a variety of anticancer drugs, and the results of these have been reported separately (Hug *et al.*, 1984*a*, & submitted). By applying the commonly used criterion of 30 or more colonies for a workable assay, twice as many tests became evaluable as a consequence of these culture modifications (Table IV).

### Discussion

Cultures of primary tumours have wide areas of application, both in the field of basic research and of clinical science. Because of the genetic instability of tumour cells, many different stem cell populations compose and maintain individual tumours. Clonogenic tumour cells are the *in vitro* counterpart of these stem cells. However, only a fraction of all tumours form colonies in semisolid cultures, and the number of colonies of those that do is generally very small. A low portion of tumour stem cells, or deficiencies of their exogenous growth support can explain these observations. Culture deficiencies can be qualitative and quantitative, since growth factors are generally mediated through receptors that are only expressed during certain phases of the cell cycle.

Established cell lines have origin and self-renewal ability in common with tumour stem cells. We reasoned that if culture deficiencies are a causative factor for the limited *in vitro* growth of tumour stem cells, these conditions could be improved using established cell lines as a tool to identify the nutrient requirements of primary tumours. To this end, we selected a group of breast tumour cell lines with diverse growth characteristics to cover the range of biologic behaviour of the primary tumour type. We then defined type and effective dose-range of nutrients required for their clonogenic growth by testing a series of defined and non-defined growth factors.

Our findings indicate that clonogenic tumour growth can be influenced by exogenous nutritional support, and that therefore a low proportion of stem cells is not the only cause for the observed low plating efficiency of primary tumours.

Certainly, our culture modifications led to an only modest improvement of the clonogenic growth of both established and primary tumour cells. However, we did not intend to optimize culture conditions for primary breast tumours. We merely tested the feasibility and validity of an alternate approach to improve culture conditions for primary tumours using breast tumours as an example. To assess the biologic relevance of these *in vitro* recovered clonogens, we also determined their inherent sensitivities to anticancer drugs. A concordance of *in vivo* and *in vitro* findings of 70% (Hug *et al.*, 1984*a*) indicates that, in fact, a meaningful relationship does exist between stem cells and clonogenic cells of primary tumours.

We conclude that deficient culture conditions are a contributary cause of the low plating efficiency of primary tumours and that these deficiencies can in part be identified by defining the growth requirements of established tumour cell lines of the same tumour type.

We would like to thank Dr. R. Cailleau and Dr. H. Soule for their kindness in providing the cell lines and Dr. M. Beran for critical review of the manuscript. We also thank L. Shepard for her excellent assistance in preparing the manuscript.

This research was supported in part by the Susan K. Komen Research Foundation Fund and Grant No. CA23272 from the National Cancer Institute.

### References

BRADLEY, T.R., METCALF, D. & RUSH, Y. (1966). The growth of mouse bone marrow cells *in vitro*. *J. Exp. Biol. Med. Sci.*, **44**, 287.

CAILLEAU, R. & REEVES, W.T., Jr. (1975). Human pleural effusions as a source of breast carcinoma cell lines for tissue culture. *TCA Manual*, **1**, 91.

COURTENAY, V.D. & MILLS, J. An *in vitro* colony assay for human tumours grown in immune-suppressed mice and treated *in vivo* with cytotoxic agents. *Br. J. Cancer*, **37**, 261.

HAMBURGER, A.W. & SALMON, S.E. (1977*a*). Primary bioassay of human tumor stem cells. *Science*, **197**, 461.

HAMBURGER, A.W. & SALMON, S.E. (1977*b*). Primary bioassay of human myeloma stem cells. *Clin. Invest.*, **60**, 846.

HUG, V., DREWINKO, B., SPITZER, G. & BLUMENSCHEIN, G. (1983). Effect of diethylaminoethyl-dextran on colony formation of human tumor cells in semisolid suspension cultures. *Cancer Res.*, **43**, 210.

HUG, V., THAMES, H., BLUMENSCHEIN, G., SPITZER, G. & DREWINKO, B. (1984*a*). Normalization of *in vitro* sensitivity testing of human tumor clonogenic cells. *Cancer. Res.*, **44**, 923.

HUG, V., THAMES, H., BLUMENSCHEIN, G., SPITZER, G. & DREWINKO, B. (1984*b*). Use of normalized drug sensitivities of human breast tumors for clinical correlations and for comparison of drug activities. In: *Human Tumor Clonong*, (Eds. Salmon & Trent). p. 573.

OSBORNE, C.K. & LIPPMANN, M.E. (1978). Human breast cancer in tissue culture: The effects of hormones. In: *Breast Cancer, Advances in Research and Treatment*, (Ed. McGuire). p. 103.

SANDBACH, J., VONHOFF, D.D., CLARK, G. & 3 others. (1982). Direct cloning of human breast cancer in soft agar culture. *Cancer*, **60**, 1215.

SIKIC, B.I. & TABER, R.L. (1981). Human tumor clonogenic assays. An overview. *Cancer Chemother. Pharmacol.*, **6**, 201.

SIRBASKU, D.A. (1978). Estrogen induction of growth factors specific for hormone-responsive mammary, pituitary and kidney tumor cells. *Proc. Natl Acad. Sci.*, **75**, 3786.

SOEHNLEN, B., YOUNG, L. & LIU, R. (1980). Standard laboratory procedures for *in vitro* assay of human tumor stem cells. In: *Cloning of Human Tumor Stem Cells*, (Ed. Salmon). p. 331.

SOULE, H.D., VAZQUEZ, J., LONG, A. & 2 others. (1973). A human cell line from a pleural effusion derived from a breast carcinoma. *J. Natl. Cancer Inst.*, **51**, 1409.

TODARO, G.J. & FRYLING, C. & DELARCO, J.E. (1980). Transforming growth factors produced by certain human tumor cells: Polypeptides that interact with epidermal growth factor receptors. *Proc. Natl. Acad. Sci.* **77**, 5258.

VONHOFF, D.D., COWAN, J., HARRIS, G. & REISDORM, F. (1981). Human tumor cloning: Feasibility and clinical correlations. *Cancer Chemother. Pharmacl.* **6**, 265.